# Sex differences in the association of phase angle and lung cancer mortality

**DOI:** 10.3389/fnut.2022.1061996

**Published:** 2022-12-22

**Authors:** Jinyu Shi, Hailun Xie, Guotian Ruan, Yizhong Ge, Shiqi Lin, Heyang Zhang, Xin Zheng, Chen’an Liu, Mengmeng Song, Tong Liu, Xiaowei Zhang, Ming Yang, Xiaoyue Liu, Qi Zhang, Li Deng, Xin Wang, Hanping Shi

**Affiliations:** ^1^Department of Gastrointestinal Surgery, Beijing Shijitan Hospital, Capital Medical University, Beijing, China; ^2^Department of Clinical Nutrition, Beijing Shijitan Hospital, Capital Medical University, Beijing, China; ^3^Beijing International Science and Technology Cooperation Base for Cancer Metabolism and Nutrition, Beijing, China; ^4^Key Laboratory of Cancer FSMP for State Market Regulation, Beijing, China; ^5^Department of Colorectal Surgery, Cancer Hospital of the University of Chinese Academy of Sciences, Zhejiang Cancer Hospital, Hangzhou, China

**Keywords:** phase angle, prognostic, sex differences, lung cancer, mortality

## Abstract

**Background:**

Lung cancer is a lethal malignant tumor that is common worldwide and is associated with a high incidence of malnutrition. Phase angle (PA) is a simple, objective, and non-invasive indicator of body composition that has increasingly attracted attention as an indicator of the nutritional status and prognosis of patients with malignant tumors. This study aimed to investigate the association between the PA and overall survival in patients with lung cancer.

**Methods:**

This study prospectively analyzed 804 lung cancer patients in the Investigation on Nutrition Status and its Clinical Outcome of Common Cancers (INSCOC) project from 40 hospitals in China. We used a restricted cubic spline to analyze the sex-specific association between PA and mortality in men and women with lung cancer. Cox regression analysis was used to evaluate the independent association between PA and mortality in men and women. Sensitivity analysis was performed. The Kaplan–Meier method was used to evaluate the survival of patients with high and low PA values.

**Results:**

There was an L-shaped association between PA and survival in both men and women with lung cancer (*p* = 0.019 and *p* = 0.121, respectively). Kaplan–Meier survival analysis suggested that patients with a high PA showed a better survival than patients with a low PA (*p* = 0.007 for men and *p* < 0.001 for women). Multivariate-adjusted Cox regression analysis showed that PA was an independent risk factor for mortality in men (HR = 0.79, 95% CI = 0.65–0.95, *p* = 0.015), but not in women (HR = 0.83, 95% CI = 0.67–1.04, *p* = 0.105).

**Conclusion:**

Phase angle is an independent risk factor for the mortality of male lung cancer patients. However, its role in predicting the mortality of female lung cancer patients seems to be limited.

## 1 Introduction

Lung cancer is a lethal malignancy that is common worldwide. According to Global Cancer Statistics 2020, lung cancer ranks first in both incidence and mortality among male tumors, and third in incidence and second in mortality among female tumors (1). According to the latest data from the National Cancer Center of China, lung cancer was the most common malignancy and the leading cause of cancer-related mortality in 2016 (2). It has been estimated that 8,28,100 new lung cancer patients and 6,57,000 lung cancer-related deaths occurred China in 2016, indicating that it poses a marked threat to the lives and health of people (2).

Previous studies have shown that the incidence of malnutrition in patients with lung cancer is as high as 45–69% (3). Malnutrition not only prolongs the length of hospitalization and increases the cost of hospitalization, but also has a marked impact on the treatment efficacy and prognosis of patients. Therefore, exploring simple and effective nutrition-related indicators to predict the prognosis of patients with lung cancer, guide clinical decision-making, and reduce lung cancer-related mortality has become an urgent problem.

Previously, nutritional screening and assessment tools, such as body mass index (BMI), dual-energy X-ray absorptiometry (DXA), computed tomography (CT), have been used to evaluate the nutritional status and predict the prognosis of patients with malignant tumors, and was successful to a certain extent (4, 5). However, there are still some deficiencies in these indicators, such as the high technical requirements and radiation damage associated with DXA, the cost and unsuitability for short-term repeated applications of CT, and the inability of BMI to distinguish between muscle and fat.

As a technology for measuring body composition using electrical methods, bioelectrical impedance analysis (BIA) utilizes the differences in electrical conductivity characteristics of various components of the human body to detect intracellular fluid resistance, extracellular fluid resistance, and cell membrane capacitance, by providing constant current signals and different electrical frequencies. BIA can be used to measure patients’ body composition and to assess their nutritional status in clinical practice (6, 7). However, BIA data are complex, and it is difficult to perform clinical analysis directly.

Phase angle (PA) is a nutritional status evaluation index derived from BIA, which can evaluate the integrity of cell membranes and the distribution of intracellular and extracellular water. PA is simple, convenient, non-invasive, time-saving, relatively objective, and is strongly sensitive to the nutritional status of patients (8–11). Previous studies have shown that the PA value can be used for the early assessment of malnutrition and that this value is associated with prognosis in patients with non-small cell lung cancer, breast cancer, pancreatic cancer, ovarian cancer, colorectal cancer, and other diseases (12–15). Due to physiological differences, there are obvious differences in body composition between men and women (16). It is unclear whether sex differences affect the role of PA in the prognostic evaluation of lung cancer patients. Therefore, it is unreasonable to use PA to assess the prognosis of patients uniformly.

Thus, the aim of this study was to explore sex differences in the association between PA and lung cancer mortality, to provide a reference for early clinical screening to identify malnourished patients and to assist clinicians in clinical decision-making and in improving patient outcomes.

## 2 Materials and methods

### 2.1 Study population

This was a prospective, multicenter study. The enrolled population was obtained from the Investigation on Nutrition Status and its Clinical Outcome of Common Cancers (INSCOC) project, which contains the clinical data of cancer patients from more than 40 hospitals in China, from June 2012 to June 2021. Patients were enrolled in the study if they were diagnosed with lung cancer, were older than 18 years, and underwent BIA examination. In this study, 1,003 lung cancer patients who underwent BIA examination in the INSCOC project were screened, of which 199 patients were excluded because of incomplete clinical data or survival data, and the remaining 804 patients were included in the final data analysis (Supplementary Figure 1).

This study was approved by the institutional review boards of all participating institutions. Written informed consent for the clinical data to be used in the clinical study was obtained from the enrolled patients.

### 2.2 Clinicopathological variables

After admission, the general clinical characteristics, laboratory biochemical indices, anthropometric measurements, pathological types, and stages were recorded in detail and accurately. General clinical characteristics included age, sex, underlying diseases, smoking, and drinking. Laboratory biochemical indicators included white blood cells, neutrophils, lymphocytes, platelets, hemoglobin, aspartate aminotransferase, alanine aminotransferase, total protein, albumin, total bilirubin, direct bilirubin, triglycerides, and cholesterol. Anthropometric indices mainly included height, weight, and triceps skinfold thickness The pathological types and stages of the tumors were recorded based on the pathological diagnosis and radiography data.

### 2.3 Measurement of BIA

Bioelectrical impedance analysis was performed on an empty stomach or 2 h after food intake using the body composition analyzer InBody S10 (Biospace, Seoul, Korea). Patients were placed in the supine position, arms and legs were naturally abducted on both sides of the body, with the back of the hand facing upward, and the fingers were naturally stretched. Two electrodes were placed in contact with the patients’ hands and feet. By detecting the impedance values of the human body under different frequency currents, the instrument automatically analyzed and calculated the relevant indicators of human body composition, including PA, extracellular water, intracellular water, body fat mass, muscle mass, and lean body mass. PA was calculated by using the following equation: PA (°) = arctan (Xc/R) × (108/π).

### 2.4 Follow-up and outcomes

All patients were followed-up continuously, and survival data were recorded. Follow-up was continued until patients died or were lost to follow-up. The primary outcome was overall survival (OS), defined as the interval between the time of pathological diagnosis and death or the day of the last follow-up. The secondary outcomes included the Karnofsky performance status (KPS) score, Nutritional Risk Screening 2002 (NRS-2002) score, Patient-Generated Subjective Global Assessment (PG-SGA) score, cachexia, and admission 30 days post-admission survival outcome. The KPS score was used to reflect general well-being and abilities of daily life, and the NRS-2002 and PG-SGA scores were used to reflect patients’ nutritional status. The diagnosis of cachexia was based on the 2011 International Consensus Framework (17).

### 2.5 Statistical analysis

Continuous variables were expressed as mean ± standard deviation or median (interquartile range). Continuous variables with a normal distribution were subjected to Student’s *t*-test, and continuous variables without a normal distribution were subjected to the Mann–Whitney *U* test. Categorical variables are expressed as frequencies or percentages, and the χ^2^ test or Fisher’s exact test was used. The sex-specific optimal cut-off values of PA were calculated with the “maxstat” package by an application “Evaluate Cutpoints” in R software. Patients were divided into high- and low-PA groups based on sex-specific optimal cut-off values. Three models were established: model a was not adjusted, model b was adjusted for age, TNM stage, and BMI, and model c was adjusted for age, TNM stage, BMI, smoking, alcohol consumption, diabetes mellitus, hypertension, coronary heart disease, chemotherapy, radiotherapy, and surgery. Multivariate Cox regression analysis was used to identify the independent significance of PA in mortality of patients with lung cancer. A restricted cubic spline (RCS) was used to assess sex-specific differences between PA and patients with lung cancer. The Kaplan–Meier method was applied to analyze the OS of men and women in the high- and low-PA groups, and the log-rank test was used for comparison between groups. Logistic regression models were used to assess the association between PA and the KPS score, NRS-2002 score, PG-SGA score, cachexia, and admission 30 days survival outcomes. *P* < 0.05 was considered statistically significant. All statistical analyses were performed using R software (version 4.2.1).

## 3 Results

### 3.1 Patient population

A total of 804 patients with lung cancer who underwent BIA were included in the analysis. There were 494 men (61.44%) and 310 women (38.56%), with a mean age of 60.60 ± 8.98 years. Of these, 435 patients (54.10%) were diagnosed with adenocarcinoma, 179 (22.26%) with squamous carcinoma, 160 (19.90%) with small cell lung cancer (SCLC), and 30 (3.73%) with other pathological types of lung cancer. There were 47 patients (5.85%) with stage I, 121 (15.05%) with stage II, 223 (27.74%) with stage III, and 413 (51.37%) with stage IV disease. The baseline demographic and clinicopathological characteristics stratified by sex are shown in Supplementary Table 1.

Supplementary Figure 2 shows that men, patients younger than 65 years, patients with a lower BMI, and patients with a lower tumor stage were more likely to have higher PA values, although the differences were not statistically significant. The results of Spearman’s rank correlation test between PA and age, tumor stage, PG-SGA score, KPS score, NRS-2002 score, and global quality of life (QoL) score are shown in Supplementary Figure 3. PA was significantly correlated with age (men, *R* = −0.46, *p* < 0.001; women, *R* = −0.24, *p* < 0.001) and NRS-2002 score (men, *R* = −0.25, *p* < 0.001; women, *R* = −0.15, *p* < 0.001). There was no correlation between PA and tumor stage (men, *R* = −0.06, *p* = 0.20; women, *R* = −0.04, *p* = 0.46), PG-SGA score (men, *R* = 0.00, *p* = 0.99; women, *R* = −0.01, *p* = 0.86), KPS score (men, *R* = 0.05, *p* = 0.26, women, *R* = 0.12, *p* = 0.04), and global quality of life score (men, *R* = −0.09, *p* = 0.06; women, *R* = −0.11, *p* = 0.05).

### 3.2 Sex differences in the association of continuous PA with OS

**FIGURE 1:**
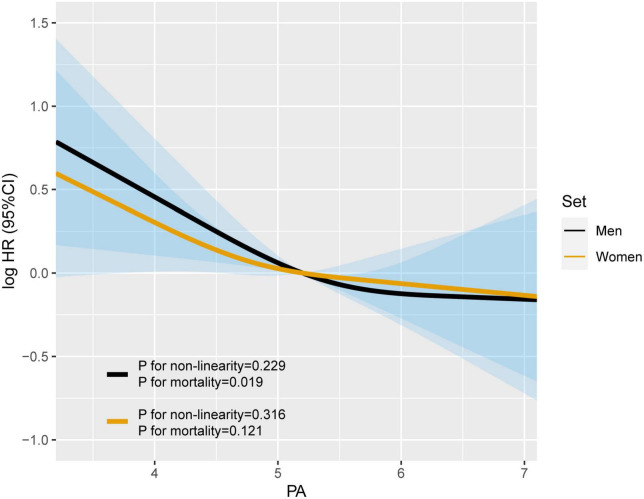
The sex differences in the association of phase angle (PA) and all-cause mortality in patients with lung cancer. Model c: Adjusted for age, TNM stage, BMI, smoking, alcohol drinking, diabetes mellitus, hypertension, coronary heart disease, chemotherapy, radiotherapy, and surgery.

**FIGURE 2:**
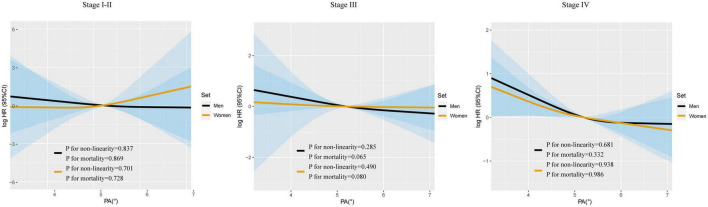
The sex-specific association between PA and all-cause mortality in patients with lung cancer based on TNM stage. Model c: Adjusted for age, TNM stage, BMI, smoking, alcohol drinking, diabetes mellitus, hypertension, coronary heart disease, chemotherapy, radiotherapy, and surgery.

Restricted cubic spline were generated to assess the sex-specific relationship between PA and mortality of patients with lung cancer. After adjusting for age, TNM stage, BMI, smoking, alcohol consumption, diabetes mellitus, hypertension, coronary heart disease, chemotherapy, radiotherapy, and surgery, we observed an L-shaped association between PA and survival in both men and women (Figure 1). PA had no statistically significant association with mortality in female lung cancer patients (p for mortality = 0.121), but was significantly associated with mortality in male patients (p for mortality = 0.019). In addition, RCS models were constructed for lung cancer patients with stage I–II, III, and IV (Figure 2). The RCS showed that there was still a tendency for sex-specific differences in all stages.

### 3.3 Sex-specific optimal cut-off values and Kaplan–Meier curves

The sex-specific optimal cut-off values for PA in terms of OS were 5.1° for men and 4.1° for women (Supplementary Figure 4). According to the above cut-off values, 190 men and 51 women were diagnosed with low-PA, and 304 men and 259 women were diagnosed with high-PA. Then, we established Kaplan–Meier survival curves for men and women with high- and low-PA values, respectively. As shown in Supplementary Figure 2 (right panels), the survival of patients with high-PA values were better than that of patients with low-PA values in both men and women (*p* = 0.007 and *p* < 0.0001, respectively).

In addition, we further established Kaplan–Meier survival curves for lung patients with high- and low-PA values in stages I–II, III, and IV (Supplementary Figure 5). The results showed that the OS of patients with high PA tended to be longer than that of patients with low PA, regardless of stage. Among men with stage III tumors and women with stage IV tumors, the OS was significantly longer in patients with high-PA than in those with low-PA values (*p* = 0.007, *p* < 0.001, respectively).

### 3.4 Sex-specific association between PA and OS

Univariate Cox regression analysis showed that continuous PA was significantly associated with mortality in men with lung cancer (*p* = 0.015) (Table 1). After adjusting for age, TNM stage, BMI, smoking, alcohol consumption, diabetes mellitus, hypertension, coronary heart disease, chemotherapy, radiotherapy, and surgery, PA was identified as an independent risk factor for mortality in men (*p* = 0.015). Similarly, when PA was used as a binary or quartile variable, the results of the multivariate Cox regression analyses continued to identify low PA as an independent risk factor for OS in men.

**TABLE 1:** Cox regression analyses for the associations between phase angle (PA) and all-cause mortality in patients with lung cancer.

Men						
**PA**	**Model a**	***P*** **-value**	**Model b**	***P*** **-value**	**Model c**	***P*** **-value**
Continuous	0.81 (0.68–0.96)	0.015	0.79 (0.66–0.95)	0.015	0.79 (0.65–0.95)	0.015
Cutoff value		0.007		0.025		0.027
C1 (≤5.1°)	Ref		Ref		Ref	
C2 (>5.1°)	0.68 (0.51–0.90)		0.70 (0.52–0.96)		0.70 (0.51–0.96)	
**Quartiles**						
Q1 (<4.8°)	Ref		Ref		Ref	
Q2 (4.8°–5.4°)	0.77 (0.52–1.13)	0.185	0.77 (0.52–1.13)	0.183	0.75 (0.50–1.12)	0.160
Q3 (5.4°–6.0°)	0.65 (0.44–0.97)	0.036	0.72 (0.47–1.10)	0.125	0.70 (0.46–1.08)	0.105
Q4 (≥6.0°)	0.61 (0.41–0.91)	0.015	0.58 (0.38–0.90)	0.014	0.59 (0.38–0.91)	0.019
p for trend		0.010		0.017		0.021
**Women**						
Continuous	0.78 (0.62–0.98)	0.029	0.80 (0.64–1.00)	0.049	0.83 (0.67–1.04)	0.105
Cutoff value		<0.001		<0.001		0.004
C1 (≤4.1°)	Ref		Ref		Ref	
C2 (>4.1°)	0.44 (0.29–0.67)		0.47 (0.30–0.73)		0.50 (0.31–0.80)	
**Quartiles**						
Q1 (<4.3°)	Ref		Ref		Ref	
Q2 (4.3°–4.8°)	0.83 (0.52–1.34)	0.454	0.83 (0.51–1.34)	0.442	0.90 (0.54–1.49)	0.676
Q3 (4.8°–5.4°)	0.41 (0.23–0.71)	0.001	0.42 (0.24–0.73)	0.002	0.44 (0.24–0.79)	0.006
Q4 (≥5.4°)	0.62 (0.38–1.01)	0.056	0.61 (0.36–1.04)	0.068	0.66 (0.38–1.12)	0.131
p for trend		0.010		0.014		0.039

Model a: no adjusted.

Model b: adjusted for age, TNM stage, and BMI.

Model c: adjusted for age, TNM stage, BMI, smoking, alcohol drinking, diabetes mellitus, hypertension, coronary heart disease, chemotherapy, radiotherapy, and surgery.

In women, the univariate Cox regression analysis showed that continuous PA was significantly associated with survival (*p* = 0.029) (Table 1). However, after adjusting for age, TNM stage, BMI, smoking, alcohol consumption, diabetes mellitus, hypertension, coronary heart disease, chemotherapy, radiotherapy, and surgery, PA was not an independent prognostic factor for mortality in women (*p* = 0.105). Interestingly, when PA was used as a binary or quartile variable, multivariate analyses identify low PA as an independent risk factor for mortality in women.

### 3.5 Sensitivity analysis of the relationship between PA and mortality

After excluding patients with severe underlying diseases (including chronic obstructive pulmonary disease and cachexia), both univariate and multivariate Cox regression analyses showed that continuous PA was an independent risk factor for mortality in men with lung cancer (*p* = 0.023 and *p* = 0.010, respectively) (Supplementary Table 2), but not in women. In addition, we obtained similar results when we excluded patients with short term deaths (within 30 days).

### 3.6 Association between PA and secondary outcomes

In this study, 748 patients (460 men and 288 women) had a KPS score ≥ 80 points. Of the 202 patients with cachexia, 125 were men and 77 were women. Seven patients, including 5 men and 2 women, died within 30 days. The NRS-2002 score was ≥3 points in 174 patients (108 men and 66 women). PG-SGA scores were ≥4 points in 461 patients (288 men and 173 women).

Logistic regression analysis showed that PA was significantly correlated with KPS score ≥ 80 points (men, *p* < 0.001; women, *p* < 0.001), cachexia (men, *p* < 0.001; women, *p* < 0.001), admission 30 days survival outcome (men, *p* = 0.012; women, *p* = 0.027), NRS-2002 score (men, *p* < 0.001; women, *p* < 0.001), and PG-SGA score (men, *p* < 0.001; women, *p* < 0.001) in both men and women (Table 2).

**TABLE 2:** Logistic regression analysis between PA and secondary outcomes.

Life function (KPS ≥ 80)						
**PA**	**Model a**	***P*** **-value**	**Model b**	***P*** **-value**	**Model c**	***P*** **-value**
Men	0.953 (0.933, 0.974)	0.019	0.956 (0.936, 0.977)	<0.001	0.947 (0.926, 0.969)	<0.001
Women	0.947 (0.926, 0.969)	<0.001	0.950 (0.929, 0.972)	<0.001	0.945 (0.923, 0.968)	<0.001
**Cachexia (yes)**						
Men	0.961 (0.948, 0.973)	<0.001	0.960 (0.948, 0.973)	<0.001	0.958 (0.946, 0.971)	<0.001
Women	0.974 (0.96, 0.988)	<0.001	0.973 (0.958, 0.987)	<0.001	0.970 (0.955, 0.985)	<0.001
**Short-term outcome (admission 30 days survival outcome)**						
Men	0.98 (0.97–0.99)	0.003	0.98 (0.96–1.24)	0.011	0.98 (0.97–1.00)	0.012
Women	0.99 (0.98–1.00)	0.063	0.99 (0.98–1.00)	0.032	0.99 (0.98–1.00)	0.027
**Malnutrition (NRS-2002 score ≥ 3)**						
Men	0.964 (0.952, 0.977)	<0.001	0.966 (0.954, 0.979)	<0.001	0.964 (0.951, 0.977)	<0.001
Women	0.965 (0.951, 0.98)	<0.001	0.966 (0.951, 0.981)	<0.001	0.962 (0.947, 0.977)	<0.001
**Malnutrition (PG-SGA ≥ 4)**						
Men	0.976 (0.965, 0.987)	<0.001	0.977 (0.966, 0.988)	<0.001	0.973 (0.961, 0.985)	<0.001
Women	0.973 (0.959, 0.987)	<0.001	0.974 (0.960, 0.988)	<0.001	0.968 (0.953, 0.983)	<0.001

Model a: no adjusted.

Model b: adjusted for age and TNM stage.

Model c: adjusted for age, TNM stage, surgery, radiotherapy, chemotherapy, hypertension, diabetes, smoking, drinking, and family history.

## 4 Discussion

The results of this study showed that PA is an independent risk factor for mortality in men with lung cancer. Although low PA is associated with a poor prognosis in women with lung cancer, PA was not identified as an independent risk factor for female mortality. Thus, PA is a more significant predictor of prognosis in men than in women with lung cancer. Even after excluding patients with severe underlying diseases or short-term deaths, sex differences in PA persisted in predicting prognosis in patients with lung cancer. In addition, we clarified that the optimal cut-off values of PA for men and women with lung cancer were 5.1° and 4.1°, respectively. Interestingly, there were no sex differences in the association of PA with patients’ KPS score, NRS-2002 score, PG-SGA score, short-term outcomes, or cachexia, and PA was significantly associated with all of these secondary outcomes. Therefore, PA can be used to assess the ability to perform activities of daily life and general well-being, nutrition, cachexia, and short-term outcomes in both men and women.

Previous studies have shown that PA is closely associated with patients’ survival. Toso et al. used an average PA of 4.5° as the cut-off value for patients with advanced lung cancer and found that the survival of patients in the low-PA group was shorter than that in the high-PA group (18). Gupta et al. found that patients with stage IV colorectal cancer with a PA > 5.57° had better survival (14). Moreover, lung cancer patients with PA < 5.3° had shorter survival times than those with high PA (19). In the present study, the optimal cut-off values of PA were 5.1° and 4.1° in men and women with lung cancer, respectively. Consistent with the results of previous studies, it was found that the survival of men and women with PA > 5.1° and >4.1° was significantly better than that of patients with low PA. Moreover, survival analysis of male and female patients was performed based on tumor stage, and the results showed that the OS of patients with a high PA tended to be longer than that of patients with a low PA. Some randomized controlled trials have demonstrated that resistance training can improve PA levels in patients, therefore, early detection of PA levels in patients and appropriate exercise are necessary (20–22).

Malnutrition is a common manifestation in patients with lung cancer (3, 23). Malnutrition works in conjunction with inflammation and the immune status to affect the health level of the body, leading to a poor prognosis (24, 25). It is one of the main factors associated with mortality. Malnutrition is characterized by altered cell membrane integrity and fluid balance. Therefore, measurement of body composition is an important part of the overall nutritional assessment of patients with cancer. As a convenient, non-invasive, and reproducible technique, PA has increasingly been used for the assessment of body composition, nutritional status, and prognosis prediction (26, 27). However, due to physiological differences between men and women, the effect of PA on the prognosis of patients of different sexes may be different. This study investigated the relationship between PA and mortality in both men and women with lung cancer. There was a sex difference in the association between PA and lung cancer mortality, and it may thus be more applicable to the prognosis assessment of men with lung cancer.

Cells consist of conductive intracellular fluid surrounded by a cell membrane that selectively allows permeation by certain ions. The electrical properties of the extracellular and intracellular fluids are close to the resistance, and the cell membrane can be equivalent to the capacitance. Thus, PA is an indicator of the relationship between resistance and capacitance (28). Previous studies have shown that PA is correlated with muscle mass and strength in cancer patients (29). Compared with women, men have a higher proportion of muscle and a higher proportion of water (including intracellular and extracellular water), so PA can more sensitively reflect changes in male body composition. Women generally have a higher percentage of body fat than men. Therefore, when evaluating the prognosis of women with lung cancer, more attention should be paid to nutritional indicators related to body fat, such as other indicators such as triceps skinfold thickness, rather than PA (5).

Phase angle detection is non-invasive, simple, objective, and easy to implement. During the diagnosis and treatment of patients with lung cancer, PA can be assessed repeatedly and dynamically in order to predict prognosis. It can be used to identify individuals with a high risk of malnutrition and poor prognosis in the early stages of the disease. During the treatment period, more clinical attention and earlier clinical intervention should be given to patients at high risk of malnutrition to avoid ineffective anti-tumor therapy and limited survival benefits and to maximize cost-effectiveness.

This study had several limitations that must be considered. First, the population included in this study was Chinese. Considering the ethnic differences in PA, this may not be applicable to populations in other countries. Second, this study may have been affected by differences in analytical instrumentation.

In summary, this study evaluated the ability of PA to predict prognosis in men and women with lung cancer. The results showed that PA does not carry equivalent prognostic value for men and for women with lung cancer, and PA was better at predicting the prognosis of male lung cancer patients than female lung cancer patients. For men, PA should be considered when performing prognostic evaluation, but for women, other nutritional indicators should be used. As an objective, convenient, non-invasive, and reproducible indicator, PA has the potential to be used widely in clinical practice to assist clinicians in the early identification and intervention in the nutritional status of lung cancer patients, to improve the prognosis of these patients.

## Data availability statement

The original contributions presented in this study are included in the article/Supplementary material, further inquiries can be directed to the corresponding authors.

## Ethics statement

The studies involving human participants were reviewed and approved by the Institutional Review Board of each hospital (Registration number: ChiCTR1800020329). The patients/participants provided their written informed consent to participate in this study.

## Author contributions

HS: conceptualization, methodology, software, and writing—reviewing and editing. JS: data curation, writing—original draft preparation, and writing—reviewing and editing. HX: writing—reviewing. GR: visualization, investigation, and writing—reviewing. YG, XinZ, CL, MS, and TL: software and validation. SL, HZ, XiaZ, and XW: supervision. MY, XL, and LD: resources. QZ: software. All authors contributed to the article and approved the submitted version.
