# Supplementary material for: Sex differences in the association of phase angle and lung cancer mortality
Source: Front Nutr. 2022 Dec 22;9:1061996. doi: 10.3389/fnut.2022.1061996 (PMC9818340; doi:10.3389/fnut.2022.1061996)
Supplement: Supplementary file 1 [file Data_Sheet_1.pdf]

Table S1. The sex-specific baseline characteristics of the study population.

Characteristic	Overall (n=804)	Men			Women		
		PA, Low (n=190)	PA, High (n=304)	p	PA, Low (n=51)	PA, High (n=259)	p
Age, years, mean (SD)	60.60 (8.98)	64.82 (8.06)	58.03 (8.48)	<0.001	65.98 (9.34)	59.47 (8.62)	<0.001
BMI, Kg/m2, mean (SD)	23.10 (3.38)	23.13 (3.28)	22.91 (3.46)	0.486	23.14 (3.18)	23.30 (3.41)	0.753
PA, mean (SD)	5.16 (0.93)	4.53 (0.52)	5.86 (0.52)	<0.001	3.62 (0.37)	5.10 (0.90)	<0.001
Diabetes, yes, n (%)	88 (10.95)	29 (15.26)	30 (9.87)	0.098	5 (9.80)	24 (9.27)	1.000
Hypertension, yes, n (%)	175 (21.77)	36 (18.95)	56 (18.42)	0.978	14 (27.45)	69 (26.64)	1.000
Coronary heart disease, yes, n (%)	44 (5.47)	13 (6.84)	16 (5.26)	0.596	1 (1.96)	14 (5.41)	0.490
Cirrhosis, yes, n (%)	5 (0.62)	1 (0.53)	2 (0.66)	1.000	1 (1.96)	1 (0.39)	0.744
Chronic hepatitis, yes, n (%)	19 (2.36)	1 (0.53)	8 (2.63)	0.175	3 (5.88)	7 (2.70)	0.459
Chronic kidney disease, yes, n (%)	15 (1.87)	3 (1.58)	7 (2.30)	0.820	1 (1.96)	4 (1.54)	1.000
COPD (%)	9 (1.12)	2 (1.05)	3 (0.99)	1.000	2 (3.92)	2 (0.77)	0.253
Smoking, yes, n (%)	492 (61.19)	161 (84.74)	238 (78.29)	0.099	21 (41.18)	72 (27.80)	0.082
Drinking, yes, n (%)	180 (22.39)	64 (33.68)	110 (36.18)	0.639	0 (0.00)	6 (2.32)	0.588
Pathological diagnosis, n (%)				0.001			0.102
Adenocarcinoma	435 (54.10)	68 (35.79)	153 (50.33)		29 (56.86)	185 (71.43)	
Squamous carcinoma	179 (22.26)	72 (37.89)	79 (25.99)		5 (9.80)	23 (8.88)	
SCLC	160 (19.90)	38 (20.00)	65 (21.38)		13 (25.49)	44 (16.99)	
other	30 (3.73)	12 (6.32)	7 (2.30)		4 (7.84)	7 (2.70)	
Clinical stage, n (%)				0.144			0.082
Stage I	47 (5.85)	4 (2.11)	20 (6.58)		3 (5.88)	20 (7.72)	
Stage II	121 (15.05)	32 (16.84)	54 (17.76)		1 (1.96)	34 (13.13)	
Stage III	223 (27.74)	64 (33.68)	92 (30.26)		10 (19.61)	57 (22.01)	
Stage IV	413 (51.37)	90 (47.37)	138 (45.39)		37 (72.55)	148 (57.14)	
Surgery, yes, n (%)	14 (1.74)	1 (0.53)	9 (2.96)	0.123	0 (0.00)	4 (1.54)	0.830
Chemotherapy, yes, n (%)	550 (68.41)	142 (74.74)	229 (75.33)	0.967	34 (66.67)	145 (55.98)	0.209
Radiotherapy, yes, n (%)	21 (2.61)	3 (1.58)	9 (2.96)	0.503	1 (1.96)	8 (3.09)	1.000
WBC (median (IQR))	6.34 (5.05, 8.06)	6.82 (5.16, 8.67)	6.48 (5.46, 8.16)	0.405	5.84 (4.37, 7.64)	5.99 (4.86, 7.56)	0.503
PLT (median (IQR))	238.00 (196.00, 297.00)	234.50 (195.00, 300.75)	235.50 (194.75, 281.50)	0.302	232.00 (172.00, 304.00)	249.00 (203.50, 313.00)	0.170

Hb (median (IQR))	134.00 (122.00, 145.00)	130.00 (118.00, 142.75)	142.00 (131.00, 151.00)	<0.001	117.00 (105.00, 131.00)	130.00 (121.00, 138.00)	<0.001
NLR (median (IQR))	2.43 (1.67, 3.65)	2.77 (1.83, 4.25)	2.39 (1.70, 3.42)	0.005	3.01 (1.85, 5.73)	2.19 (1.56, 3.17)	0.001
ALI (median (IQR))	36.97 (22.74, 55.17)	30.18 (18.53, 48.48)	37.89 (24.99, 57.46)	0.001	25.06 (14.46, 46.32)	42.95 (28.24, 61.62)	<0.001
Total protein (median (IQR))	69.10 (65.07, 73.10)	68.25 (64.00, 72.00)	69.20 (65.65, 73.20)	0/039	65.20 (59.60, 70.05)	69.90 (66.50, 73.80)	<0.001
Albumin (median (IQR))	38.70 (35.70, 41.40)	37.40 (34.28, 40.20)	39.35 (37.08, 42.20)	<0.001	36.00 (31.55, 38.50)	39.30 (36.55, 41.65)	<0.001
Total bilirubin (median (IQR))	10.30 (7.50, 14.00)	9.80 (7.30, 13.40)	10.60 (7.70, 14.43)	0.137	9.60 (6.70, 12.95)	10.50 (7.80, 13.90)	0.362
Direct bilirubin (median (IQR))	2.80 (2.10, 3.70)	3.05 (2.20, 4.10)	2.90 (2.20, 3.70)	0.272	2.50 (1.80, 3.65)	2.70 (1.95, 3.45)	0.811
Cholesterin (median (IQR))	4.65 (4.00, 5.30)	4.42 (3.80, 5.05)	4.62 (3.99, 5.17)	0.028	4.75 (4.27, 5.83)	4.89 (4.22, 5.63)	0.970
Triglyceride (median (IQR))	1.33 (1.00, 1.91)	1.21 (0.86, 1.78)	1.29 (0.96, 1.89)	0.164	1.46 (1.10, 2.15)	1.44 (1.12, 1.97)	0.610
AST (median (IQR))	22.10 (17.70, 27.25)	21.35 (17.02, 26.98)	21.10 (17.80, 26.75)	0.573	23.20 (18.30, 30.10)	22.80 (18.50, 27.05)	0.569
ALT (median (IQR))	18.15 (12.70, 27.13)	19.20 (13.12, 26.55)	20.35 (14.07, 31.70)	0.057	12.70 (9.55, 21.80)	16.70 (11.40, 25.20)	0.022
Muscle mass (median (IQR))	43.90 (37.80, 50.23)	43.20 (37.42, 49.58)	44.25 (37.30, 50.60)	0.644	43.20 (38.75, 48.10)	44.20 (38.40, 50.50)	0.569
FFM (median (IQR))	46.75 (40.20, 53.10)	45.95 (39.90, 52.85)	47.05 (39.98, 53.25)	0.646	45.70 (40.65, 50.95)	47.10 (41.00, 53.40)	0.389
Hand grip strength (median (IQR))	24.70 (18.28, 33.28)	25.10 (17.58, 32.58)	24.15 (18.30, 32.62)	0.965	25.60 (17.30, 34.10)	25.40 (18.55, 34.45)	0.963
body fat mass	15.70 (11.00, 20.62)	15.50 (11.05, 20.55)	16.20 (11.15, 20.52)	0.017	15.50 (11.75, 19.95)	15.50 (10.90, 21.50)	0.893
Extracellular water	13.50 (11.70, 15.10)	13.20 (11.40, 15.00)	13.50 (11.60, 15.10)	0.048	13.40 (12.00, 14.60)	13.60 (11.80, 15.10)	0.720
Intracellular water content	21.10 (18.17, 24.20)	20.75 (17.90, 24.12)	21.50 (17.90, 24.30)	0.056	21.30 (18.75, 23.15)	21.10 (18.50, 24.25)	0.614
TSF (median (IQR))	15.00 (11.00, 22.00)	15.00 (11.00, 21.75)	15.00 (11.00, 23.00)	0.569	16.00 (11.00, 20.50)	15.00 (11.00, 22.00)	0.807
KPS score (median (IQR))	90.00 (80.00, 90.00)	90.00 (80.00, 90.00)	90.00 (80.00, 90.00)	0.862	90.00 (80.00, 95.00)	90.00 (80.00, 90.00)	0.894
PG-SGA score (median (IQR))	4.00 (2.00, 7.00)	5.00 (2.00, 7.00)	4.00 (2.00, 7.25)	0.801	5.00 (2.00, 6.00)	4.00 (2.00, 7.00)	0.814

Notes: PA, phase angle; COPD, chronic obstructive pulmonary disease; SCLC, small cell lung cancer; WBC, white blood cell; PLT, platelet; Hb, hemoglobin; NLR, neutrophil-to-lymphocyte ratio; ALI, advanced lung cancer inflammatory index; AST, aspartate aminotransferase; ALT, alanine aminotransferase; FFM, fat-free mass; TSF, triceps skinfold thickness; KPS, Karnofsky performance status; PG-SGA, Patient-Generated Subjective Global Assessment.

Table S2. The sensitivity analysis of the relationship between PA and mortality by excluding patients with severe underlying diseases.

			Men			
PA	Model a	p value	Model b	p value	Model b	p value
Continuous	0.78(0.63-0.97)	0.023	0.72(0.57-0.92)	0.010	0.72(0.56-0.92)	0.010
Cutoff value		0.012		0.071		0.076
C1 (≤5.1°)	ref		ref		Ref	
C2 (>5.1°)	0.65(0.46-0.91)		0.70(0.48-1.03)		0.71(0.48-1.04)	
Quartiles						
Q1 (<4.8°)	ref		ref		Ref	
Q2 (4.8° -5.4°)	0.74(0.46-1.17)	0.191	0.70(0.44-1.12)	0.140	0.67(0.41-1.09)	0.106
Q3 (5.4° -6.0°)	0.56(0.35-0.89)	0.014	0.63(0.38-1.05)	0.074	0.61(0.36-1.01)	0.053
Q4 (≥6.0°)	0.58(0.37-0.92)	0.019	0.52(0.31-0.86)	0.014	0.52(0.30-0.88)	0.015
P for trend		0.010		0.016		0.015
			Women			
Continuous	0.93(0.71-1.21)	0.581	0.94(0.71-1.24)	0.66	0.97(0.73-1.28)	0.811
Cutoff value		0.022		0.028		0.054
C1 (≤4.1°)	ref		ref		Ref	
C2 (>4.1°)	0.52(0.29-0.91)		0.51(0.28-0.93)		0.53(0.28-1.01)	
Quartiles						
Q1 (<4.3°)	ref		ref		Ref	
Q2 (4.3° -4.8°)	1.02(0.54-1.92)	0.945	1.00(053-1.88)	0.989	1.03(0.53-2.00)	0.936
Q3 (4.8° -5.4°)	0.53(0.26-1.09)	0.084	0.55(0.27-1.13)	0.105	0.55(0.25-1.20)	0.132
Q4 (≥5.4°)	0.77(0.41-1.46)	0.427	0.74(0.37-1.49)	0.400	0.79(0.38-1.64)	0.521
P for trend		0.207		0.198		0.301

Notes:

Model a: No adjusted.

Model b: Adjusted for age, TNM stage, BMI.

Model c: adjusted for age, TNM stage, BMI, smoking, alcohol drinking, diabetes mellitus, hypertension, coronary heart disease, chemotherapy, radiotherapy, surgery.

Table S3. The sensitivity analysis of the relationship between PA and mortality by excluding patients with short-term deaths (30-days).

			Men			
PA	Model a	p value	Model b	p value	Model c	p value
Continuous	0.84(0.70-1.00)	0.048	0.82(0.68-0.99)	0.041	0.81(0.66-0.99)	0.036
Cutoff value		0.016		0.041		0.038
C1 (≤5.1°)	ref		ref		Ref	
C2 (>5.1°)	0.70(0.53-0.94)		0.72(0.53-0.99)		0.71(0.52-0.98)	
Quartiles						
Q1 (<4.8°)	ref		ref		Ref	
Q2 (4.8°-5.4°)	0.83(0.56-1.23)	0.345	0.82(0.55-1.22)	0.331	0.80(0.53-1.20)	0.278
Q3 (5.4°-6.0°)	0.68(0.46-1.03)	0.068	0.75(0.49-1.15)	0.183	0.72(0.47-1.12)	0.147
Q4 (≥6.0°)	0.66(0.44-0.98)	0.038	0.62(0.40-0.96)	0.031	0.62(0.39-0.97)	0.035
P for trend		0.025		0.032		0.035
			Women			
Continuous	0.80(0.64-1.00)	0.052	0.83(0.67-1.03)	0.087	0.86(0.70-1.07)	0.178
Cutoff value		<0.001		0.004		0.019
C1 (≤4.1°)	ref		ref		Ref	
C2 (>4.1°)	0.47(0.31-0.72)		0.51(0.32-0.81)		0.56(0.34-0.91)	
Quartiles						
Q1 (<4.3°)	ref		ref		Ref	
Q2 (4.3°-4.8°)	0.88(0.54-1.43)	0.61	0.88(0.54-1.44)	0.613	0.97(0.58-1.63)	0.914
Q3 (4.8°-5.4°)	0.43(0.24-0.75)	0.003	0.44(0.25-0.78)	0.005	0.47(0.26-0.87)	0.015
Q4 (≥5.4°)	0.65(0.40-1.07)	0.091	0.66(0.39-1.13)	0.129	0.72(0.42-1.25)	0.246
P for trend		0.019		0.032		0.084

Notes:

Model a: No adjusted.

Model b: Adjusted for age, TNM stage, BMI.

Model c: adjusted for age, TNM stage, BMI, smoking, alcohol drinking, diabetes mellitus, hypertension, coronary heart disease, chemotherapy, radiotherapy, surgery.

Figure S1. Study design.

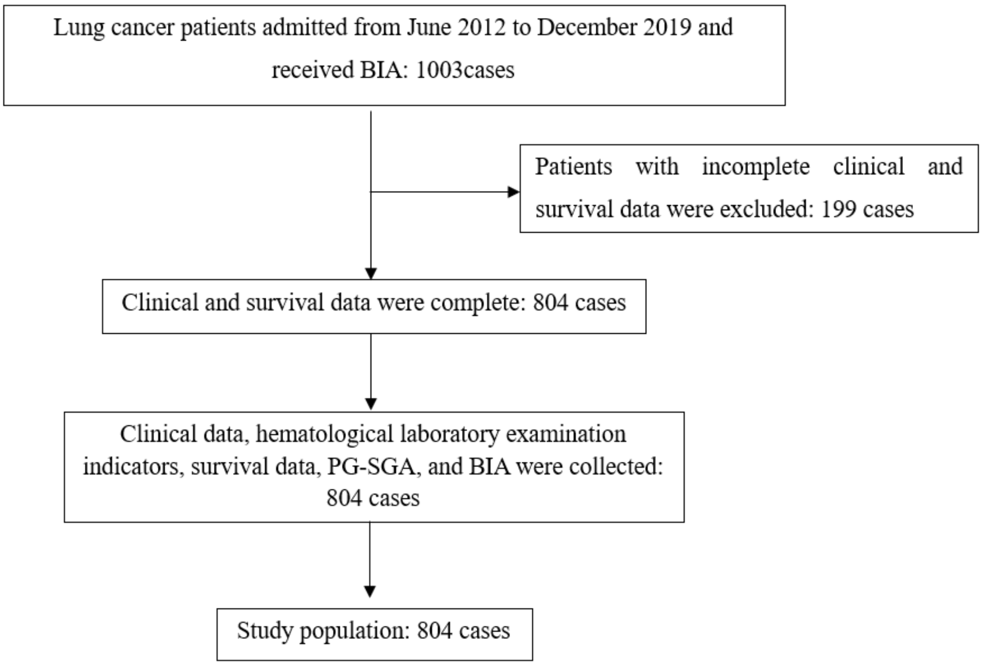

Figure S2. PA (log transformation) in different clinicopathological subgroups. ns p-value >0.05, * p-value<0.05, **** p-value<0.001.

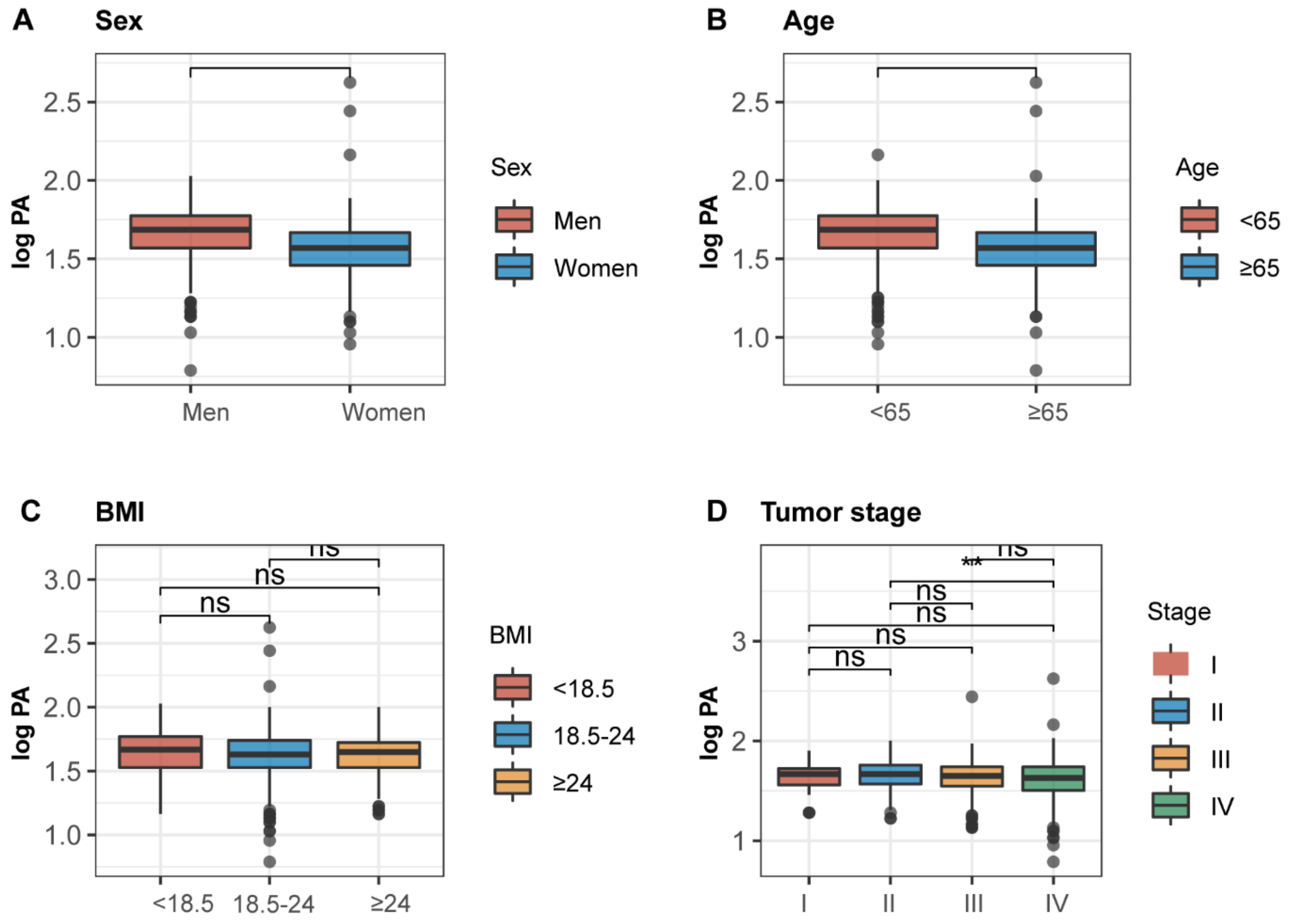

Notes: A, sex; B, age; C, BMI; D, tumor stage.

Figure S3 Correlation analysis between PA and other parameters.

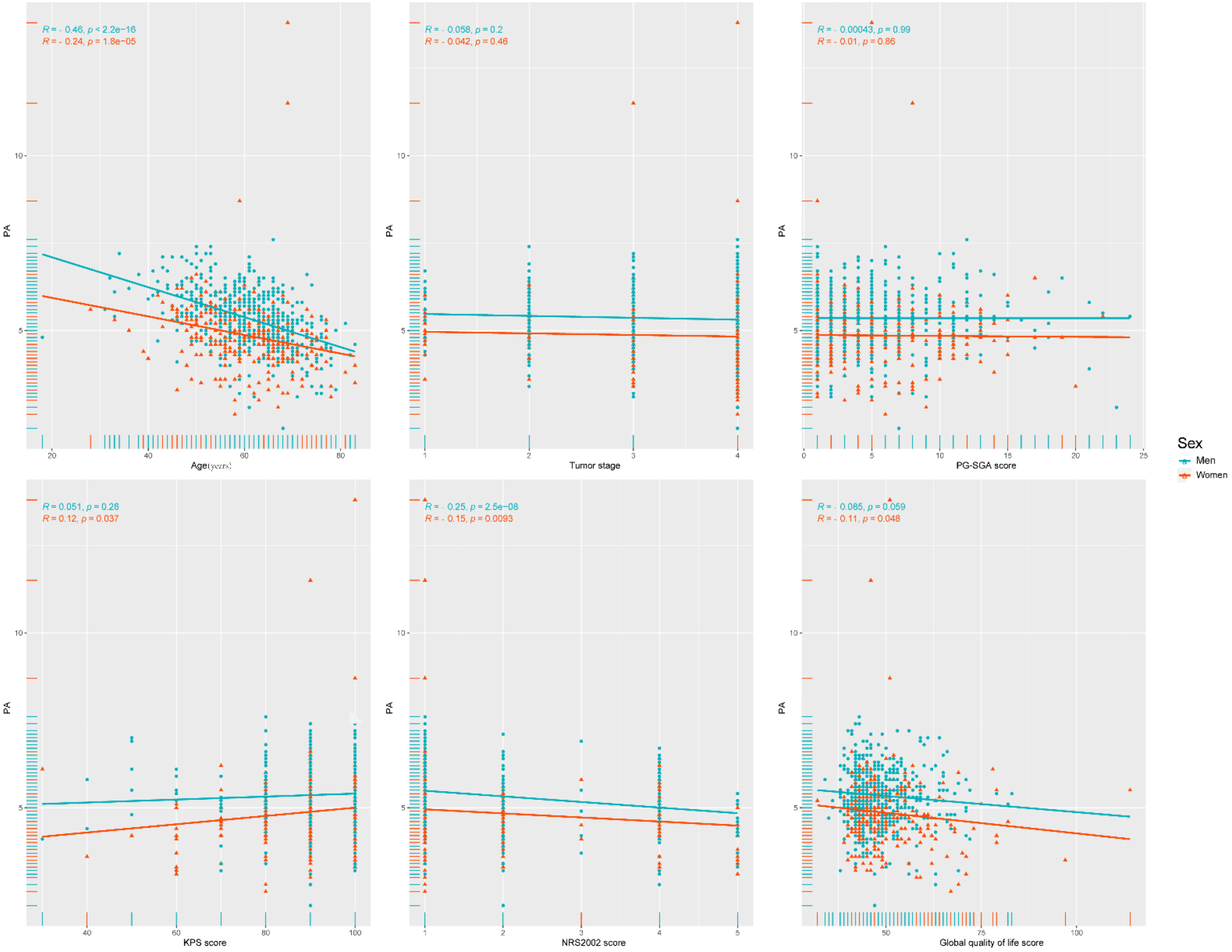

Notes: A, PA vs age; B, PA vs tumor stage; C, PA vs PG-SGA score; D, PA vs KPS score; E. PA vs NRS2002 score; F, PA vs global quality of life score.

Figure S4. Cut-off values and Kaplan-Meier curve of PA based on sex-specific (man and women) strata in patients with lung cancer.

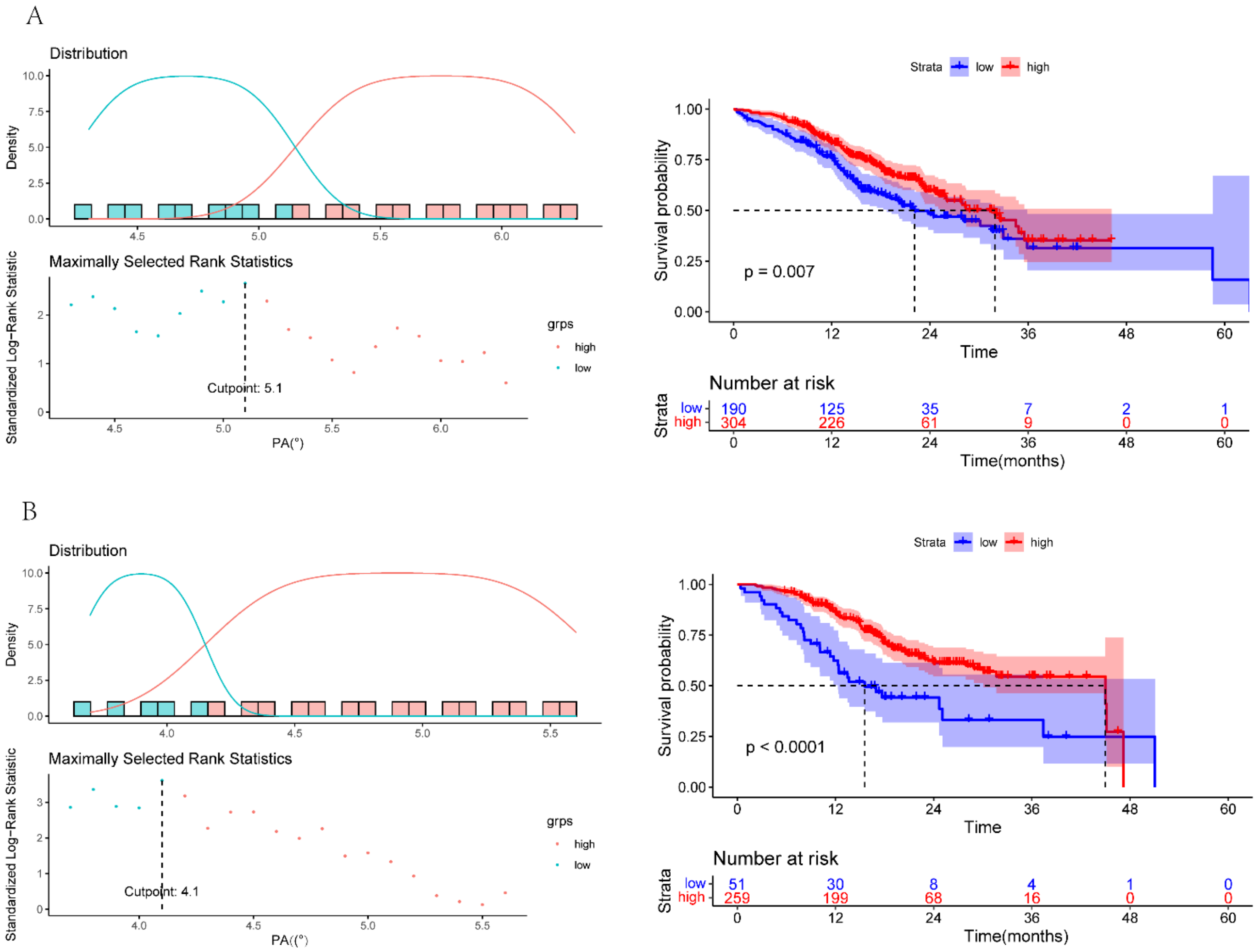

Notes: A, men; B, women

Plot of standardized log-rank statistics of PA (left panels) and the Kaplan–Meier plot according to the cut-off of PA (right panels). The optimal cut-off value of PA was 5.1° for men patients and 4.1° for women patients.

Figure S5. Sex-specific stratified survival analysis of PA based on TNM stage in patients with lung cancer.

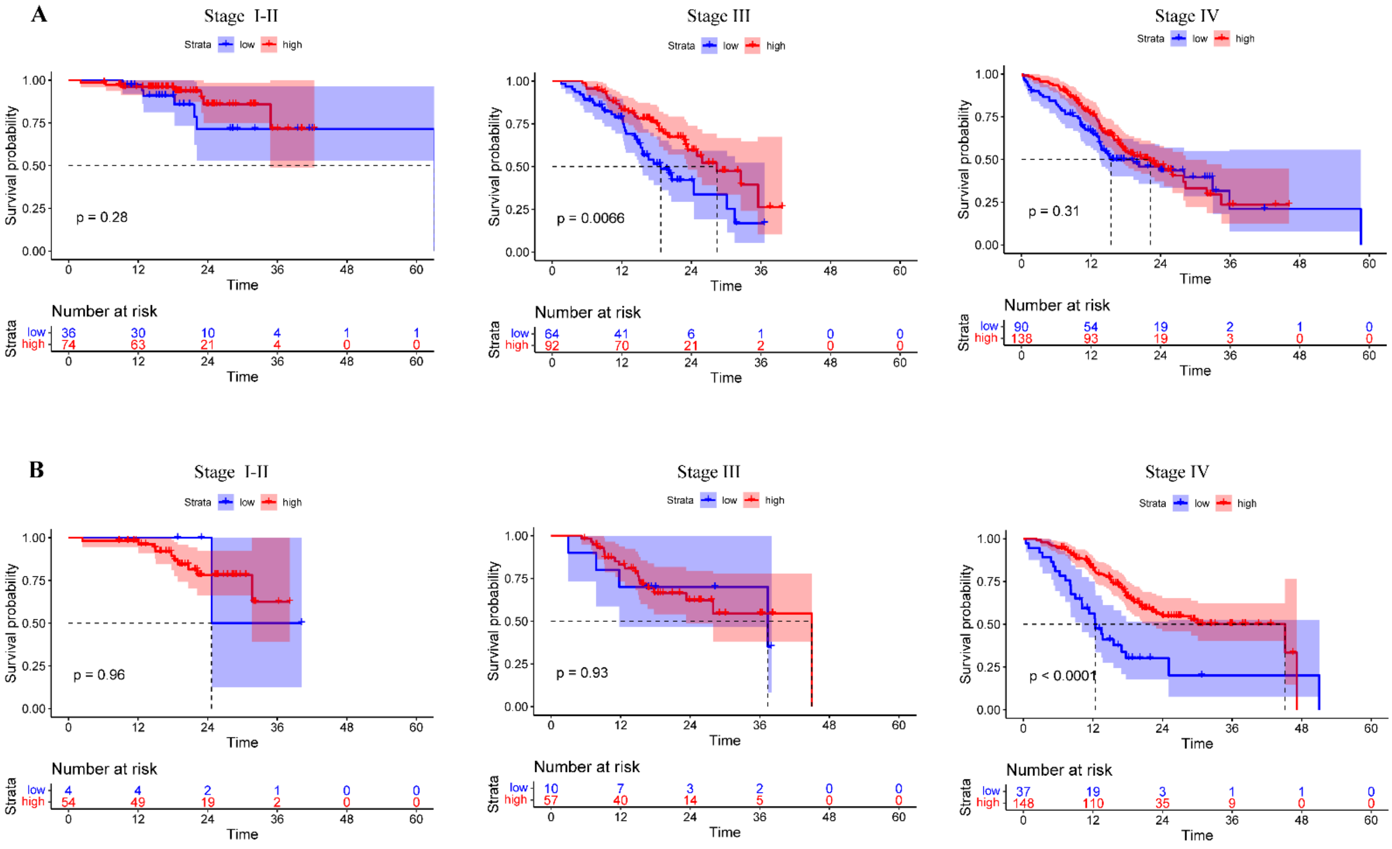

Notes: A, men; B, women.
